# Evaluating the Indications of Surgical Extraction of Impacted Lower Third Molar in the Oral Surgery Clinic, College of Dentistry, University of Sulaimani

**DOI:** 10.1155/bmri/9750143

**Published:** 2026-05-25

**Authors:** Suha Nafea Aloosi, Nizar Abdulqadir Muhammad Amin

**Affiliations:** ^1^ Department of Oral and Maxillofacial Surgery, College of Dentistry, University of Sulaimani, Sulaymaniyah, Iraq, univsul.edu.iq

**Keywords:** adjacent second molar, lower third molar, prophylactic extraction, temporomandibular disorder, temporomandibular joint

## Abstract

**Background/Objectives:**

The most commonly impacted teeth are mandibular third molars, for which their extraction results in potential complications. In our locality, there is a noticeable seek for dental evaluation detecting impacted mandibular third molars (IMTMs) by young individuals, and an evidence based on regional data from Sulaimani is required, considering possible differences in clinical practice patterns and the ongoing debate regarding whether extraction should be performed. This study is aimed at evaluating the indications for surgical extraction of the IMTM.

**Methods:**

A cross‐sectional analytical study was conducted on 120 patients referred to the Oral Surgery Clinic, College of Dentistry, University of Sulaimani, Sulaymaniyah, Iraq, for the extraction of their IMTM from January to July 2025.

**Results:**

The majority of patients were female (*n* = 74) and aged 16–25 years (64%). Some patients (*n* = 32, 26.7%) were asymptomatic and presented for a checkup; 24 (20%) had caries in the lower third molar or the adjacent second molar. Dental caries was the most common reason for extraction indication (40.7%), followed by recurrent pericoronitis (28.8%). Radiographic proximity of the IMTM to the adjacent lower second molar was the most common indication for prophylactic extraction of asymptomatic individuals (56.3%), followed by difficulty in brushing (21.9%). Prophylactic extraction was preferred due to the high and age‐related increasing incidence of caries in the third molar or the adjacent second molar (*p* = 0.037).

**Conclusions:**

While some IMTM may remain asymptomatic, others can be associated with various pathologies, including caries, pericoronitis, and root resorption of neighboring teeth.

## 1. Introduction

Prevalence of impacted third molars is 36.9% and is primarily found in the mandible (46.4%) [1]. Females experienced a higher prevalence of third molar impaction, and mesioangular is the most predominant type [2]. Many factors predispose to teeth impaction, among these are lack of space in the dental arch, which may result from discrepancy in teeth and/or jaw dimensions, abnormal position of the impacted teeth, and the density of the overlying gingival and bony tissue [3]. A shift in dietary habits is a cause for tooth impaction, as it leads to reduced space for teeth to erupt [4]. Impacted mandibular third molar (IMTM) is often associated with recurrent pericoronitis, adjacent tooth caries, and lower anterior arch crowding. Periodontal defects of adjacent molars and mandibular second molar root resorption increase the need for their extraction [5]. Recurrent pericoronitis is the most common reason for surgically removing IMTM [6].

Several indications for the extraction have been suggested, including preventing late mandibular incisor crowding. Most orthodontists and oral surgeons believed that IMTM can create a forward force component that causes crowding of the lower incisors [7]. Intraoperative complications of extracted IMTM may include bleeding, damage to adjacent teeth, injury to surrounding tissues and nerves, displacement of teeth into adjacent spaces, fracture of the mandible, and loss of support adjacent to the tooth. Postoperative complications may include swelling, pain, trismus, prolonged bleeding, localized alveolar osteitis, and paresthesia [8]. About 10% of third molar extraction patients have requested postoperative emergency appointments [9]. The decision to commit a patient to the removal of a third molar can be complex and challenging. General dentists tend to recommend removal if the third molars have not erupted [10], and it is essential to analyze the surgical difficulty before performing the procedure [11]. The application of evidence‐based medicine should be encouraged, as it enables surgeons to more accurately assess the risk–benefit ratio, anticipate potential complications, and evaluate the likelihood of encountering difficulties. Employing the best clinically relevant evidence is the most effective approach to addressing any clinical dilemma. Many authors believe that only individuals with pathologically proven third molars should have their impacted teeth surgically removed, such as unrestorable caries, periodontal pathology, cellulitis, abscess, osteomyelitis, internal or external resorption of the tooth or neighboring teeth, the need for reconstructive jaw surgery, or when a tooth is involved within the field of tumor resection [12]. There is an agreement about the removal of third molars when pathology is present; however, there is a debate regarding their prophylactic removal. Extraction is indicated in the presence of a disease associated with an IMTM, whether symptomatic or not. Many oral surgeons encourage the removal of impacted teeth as soon as their presence is confirmed [13].

According to the National Institute for Health and Care Excellence, the prophylactic removal of pathology‐free IMTM should be discontinued, and there is no reliable evidence to support a health benefit to this removal [12]. The issue of third molars and their removal is becoming an increasingly important question for teenagers and young adults. When more than a third of the population faces a decision about how best to manage impacted teeth, this places a significant burden on the healthcare sector [10]. In our locality, an increasing number of young individuals seek dental evaluation for IMTM, placing additional demands on healthcare resources. This trend likely reflects increased health awareness and a proactive approach to oral health maintenance. Accurate clinical decision‐making is crucial to reduce unnecessary procedures and prevent complications, such as inferior alveolar nerve injury or alveolar osteitis. Therefore, region‐specific evidence from Sulaimani is required, particularly in light of variations in clinical practice and the ongoing debate regarding the indications for third molar extraction. Thus, this study is aimed at analyzing the scientific indication for IMTM extraction.

## 2. Patients and Methods

### 2.1. Study Setting and Design

This cross‐sectional analytical study included 120 patients (46 males and 74 females) who presented to the Oral Surgery Clinic at College of Dentistry, University of Sulaimani, Sulaymaniyah, Iraq, to extract their IMTM from January to July 2025. Referrals were based on potential complications, interference with orthodontic treatment, or existing complications, such as infection (*n* = 88). However, some of them (*n* = 32) visited the clinic for a dental checkup. Sample size was determined by the availability of eligible cases at the time of study, consistent with STROBE guidance for observational studies.

### 2.2. Inclusion Criteria

Inclusion criteria were as follows: patients with IMTM, aged ≥ 16 years old, presenting with potential complications, interference with orthodontic treatment, or dental infection.

### 2.3. Exclusion Criteria

Participants with bleeding problems or those on chronic anticoagulants for a specific disease, or those who had chronic diseases, such as cancer, were excluded.

### 2.4. Study Protocol

After obtaining a detailed history from each patient regarding their chief complaint, they were examined by an expert senior oral and maxillofacial surgeon. The assessment includes both extraoral and intraoral examinations, as well as evaluations of hard and soft tissues, occlusion, and tooth alignment. Special focus was given to the lower third molar area, assessing soft tissue condition, eruption status, enamel/dentin integrity, and periodontal health. An orthopantomogram was required for all patients, while cone beam computed tomography (CBCT) and lateral cephalometric radiographs were performed when needed. CBCT was used to assess the proximity of the impacted tooth to adjacent teeth and the inferior alveolar nerve. At the same time, the cephalometric radiograph was typically requested to complete the evaluation. Data from the patient′s history, clinical examination, and radiographic investigations were recorded. Two expert examiners evaluated each case to determine the surgical indication based on the available evidence. A senior oral surgeon assessed all cases, with input from a specialist orthodontist or periodontologist when indicated by the patient′s symptoms or chief complaint. Patients were consulted regarding the decision to proceed with extraction or to defer the procedure, and written informed consent was obtained. Then, all cases were scheduled for surgical extraction of the lower third molar at the department′s clinic under local anesthesia.

### 2.5. Data Analysis

Analytical methods were employed in the statistical analysis using the Statistical Package for the Social Sciences (IBM SPSS, Chicago, United States; Version 29). Pearson correlation coefficient, chi‐square, and Kruskal–Wallis tests were used to assess the relationship between categorical variables. Descriptive statistics were used to summarize demographic data, while the incidence of extraction decisions was reported as frequencies and percentages. A *p* < 0.05 was considered statistically significant.

## 3. Results

The majority of patients (64%) were aged 16–25 years, 27.5% were 26–35 years, and 8.3% were ≥ 36 years. Pain was the most common complaint and reason for seeking extraction of the third molar, reported by 62 (51.7%) patients. In contrast, 32 patients (26.7%) were asymptomatic, with no esthetic or functional issues, and presented only for a routine checkup (Table 1).

**Table 1:** Patients′ chief complaints at presentation.

Variable	Frequency	Percent
Checkup	32	26.7
Discomfort	7	5.8
Esthetic	15	12.5
Pain	62	51.7
Swelling	4	3.3
Total	120	100

**Figure 1:**
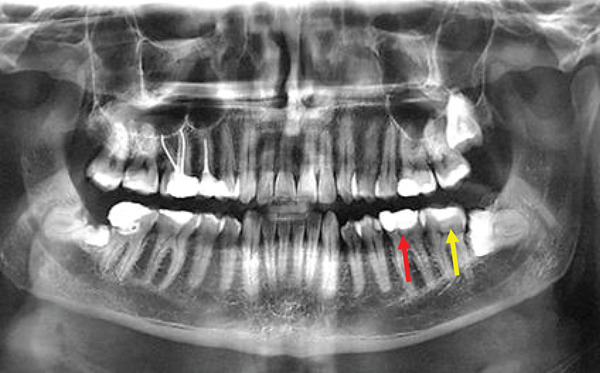
A radiological picture showing a huge filling in the right second molar and caries of the right impacted lower third molar.

**Figure 2:**
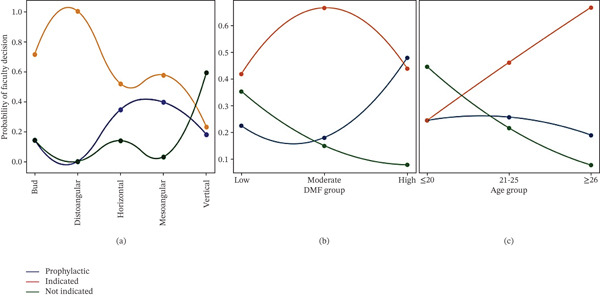
Smooth probability curves demonstrating the relationship between variables and faculty extraction decision (prophylactic, indicated, and not indicated). Curves represent smoothed probability trends across categories: (a) with tooth angulation, (b) with DMF score, and (c) with age groups (≤ 20, 21–25, and ≥ 26 years).

A team of specialized faculty dentists made the diagnosis based on clinical and radiological findings. Caries affecting the lower third molar or adjacent second molar was observed in 24 patients (20%). Among them, 19 (15.8%) showed close approximation to the second molar as indicated by the CBCT (Figure 1). Among the affected teeth with caries in this study, 41.7% were mesioangular (Figure 2), 25% vertical, and 25% horizontally impacted. Recurrent pericoronitis was identified in 21 patients (17.5%), while temporomandibular joint (TMJ) or myofascial pain was diagnosed in 17 (14.2%) cases, using standardized diagnostic criteria (such as DC/temporomandibular disorder [TMD]) to rule out primary joint pathology before recommending extraction. Also, nine (7.5%) patients reported difficulty in brushing, seven (77.8%) of them had prophylactic extractions (PEs; 21.9%) (Table 2). Among the referred patients, 59 (49.2%) had a clear indication and underwent extraction. PE was performed in 32 patients (26.7%), while extraction was deferred in 29 patients (24.2%) (Table 3).

**Table 2:** The faculty decision in indicating the extraction of the impacted third molar relation to the diagnosis.

Diagnosis	Total patients	Prophylactic extraction	Indicated extraction	Deferment of extraction	Prophylactic cases	Indicated cases	Deferred cases
	Number (percentage)				Percentage		
Normal	8 (6.7)	2 (25.0)	0 (0.0)	6 (75.0)	6.3	0.0	20.7
Caries (7 or 8)	24 (20.0)	0 (0.0)	24 (100.0)	0 (0.0)	0.0	40.7	0.0
Pericoronitis	21 (17.5)	0 (0.0)	17 (81.0)	4 (19.0)	0.0	28.8	13.8
Proximity to the lower 7	19 (15.8)	18 (94.7)	0 (0.0)	1 (5.3)	56.3	0.0	3.4
TMJ/myofascial pain	17 (14.2)	5 (29.4)	0 (0.0)	12 (70.6)	15.6	0.0	41.4
Malocclusion	13 (10.8)	0 (0.0)	10 (76.9)	3 (23.1)	0.0	16.9	10.3
Difficulty in brushing	9 (7.5)	7 (77.8)	2 (22.2)	0 (0.0)	21.9	3.4	0.0
Crowding	5 (4.2)	0 (0.0)	4 (80.0)	1 (20.0)	0.0	6.8	3.4
Periodontitis	4 (3.3)	0 (0.0)	2 (50.0)	2 (50.0)	0.0	3.4	6.9
Total	120 (100)	32 (26.7)	59 (49.2)	29 (24.2)	100	100	100

Abbreviation: TMJ, temporomandibular joint.

**Table 3:** Association between age and chance of caries occurrence.

Caries 7 or 8	Age (years)																			Total
	17	18	19	20	21	22	23	24	25	26	27	28	30	33	34	37	40	43	44	
Count	0	0	0	0	1	2	5	1	1	0	2	0	4	1	1	0	3	2	1	24
% Within diagnosis	0	0	0	0	4.2	8.3	20.8	4.2	4.2	0	8.3	0	16.7	4.2	4.2	0	12.5	8.3	4.2	100

*Note:* Caries of 7 and 8 significantly increase with age (*p* = 0.037).

Among the patients who underwent third molar extraction, caries was the most frequently reported in the third and second molars (40.7% of all indicated cases). At the same time, 21 patients (17.5%) exhibited pericoronitis associated with IMTM. Among them, 17 (81%) were scheduled for extraction due to a low probability of natural eruption, primarily influenced by age and tooth angulation. Chi‐square analysis demonstrated a significant association between the faculty extraction decision and clinical diagnosis (*χ* ^2^ = 138.5, *p* < 0.001), with caries and pericoronitis representing the most frequent diagnoses leading to extraction presence of caries (*p* < 0.001), followed by recurrent pericoronitis (28.3%), malocclusion (16.9%), crowding (6.8%), and difficulty in brushing/periodontitis (3.4% each) (Table 2). In addition, a highly significant association was also observed between tooth angulation and the extraction decision (*χ* ^2^ = 52.15, *p* < 0.001). Distoangular impactions were almost exclusively indicated for extraction, whereas mesioangular and horizontal impactions were more frequently managed by extraction, including prophylactic removal. Among mesioangular teeth, 58% were indicated for extraction, and 39% underwent prophylactic removal, representing the highest prophylactic rate. For horizontal impactions, 51.7% were indicated for extraction, and 34.5% underwent prophylactic removal, while vertically oriented teeth were predominantly managed conservatively (59%) (Figure 2).

**Figure 3:**
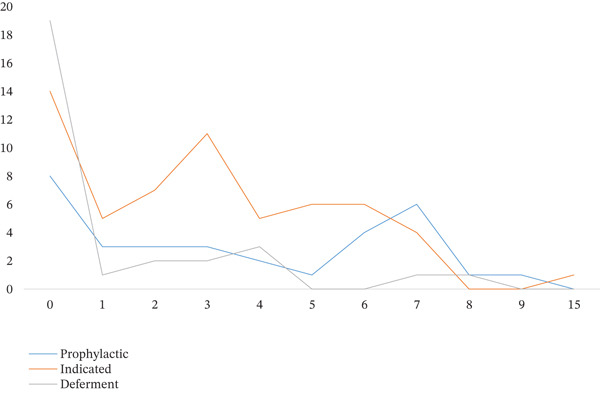
Increasing the recommendation for extracting the impacted third molar prophylactically with increasing age.

A significant difference was also observed for the DMFI (decayed, missing, and filled index) scores that were used as a proxy for patient caries experience, across extraction decisions (Kruskal–Wallis *H* = 13.61, *p* = 0.001). When specifically analyzing prophylactic removal, a significant positive correlation was found between higher DMFI and PE (Spearman *p* = 0.21 and *p* = 0.03, respectively), indicating a preventive approach when caries risk is increasing. No significant association was observed with patients′ sex (*p* = 0.32), whereas eruption status demonstrated a borderline relationship (*p* = 0.057), with partially impacted teeth more frequently indicated for extraction (Figure 2). Age group showed a borderline association with treatment decision overall (*p* = 0.069). However, analysis demonstrated that younger patients were significantly more likely to undergo PE (Mann–Whitney *U* = 785, *p* = 0.02) (Figure 3).

**Figure 4:**
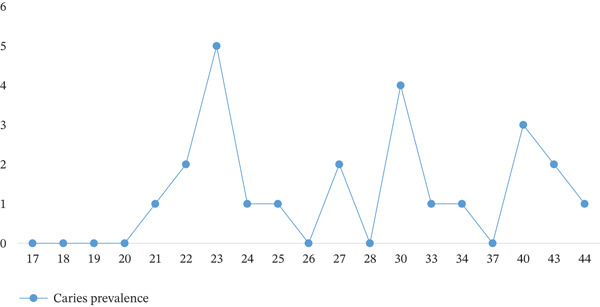
The prevalence of caries in lower impacted third molar in relation to patients′ age.

Additionally, a significant positive correlation was found between higher DMFI scores and increasing age (*p* = 0.002). The patient′s average DMFI peaks between the ages of 30 and 40. Thus, a significant association was identified between age and the presence of caries affecting both the lower third molar and the adjacent second molar (*p* = 0.037) (Table 3). The caries prevalence was low in patients < 22 years, rose in the mid‐20s to early 40s, and reached its highest in 30–44 (Figure 4). Extraction was deferred in 29 patients (24.2% of all cases). Among these, 65.5% (*n* = 19) had a favorable chance of natural eruption, leading to the decision to postpone removal. Also, three (10.3%) patients had fully impacted but asymptomatic, where immediate extraction was not considered necessary. Furthermore, in seven patients (24.1%), extraction was declined following counseling regarding the potential risk of nerve injury (Table 4).

**Table 4:** Faculty‐based criteria for decision for indicating or deferring extraction of impacted lower third molars.

Faculty decision	Asymptomatic	Caries	Eruption likely	Eruption unlikely	Orthodontic need	Orthognathic surgery	Nerve injury risk	Total
	Number (percentage)							
Prophylactic (*n* = 32)	0 (0.0)	0 (0.0)	0 (0.0)	31 (96.9)	1 (3.1)	0 (0.0)	0 (0.0)	32 (100)
Indicated (*n* = 59)	0 (0.0)	24 (40.7)	0 (0.0)	21 (35.6)	12 (20.3)	2 (3.4)	0 (0.0)	59 (100)
Deferment (*n* = 29)	3 (10.3)	0 (0.0)	19 (65.5)	0 (0.0)	0 (0.0)	0 (0.0)	7 (24.1)	29 (100)
Total (*n* = 120)	3 (2.5)	24 (20.0)	19 (15.8)	52 (43.3)	13 (10.8)	2 (1.7)	7 (5.8)	120 (100)

## 4. Discussion

This study included patients who either initially presented or were referred for lower third molar extraction and exhibited various symptoms or required orthodontic intervention. The sensation of having an impacted wisdom tooth often evokes a sense of premonition among young individuals, reflecting an increased self‐awareness and a proactive attitude toward maintaining optimal health. This awareness motivates young adults to seek dental evaluation for the detection of IMTM and to pursue prophylactic measures to prevent the complications associated with this condition.

Pain was the primary reason for clinic visits, as reported by 51.7% of our patients. This aligns with Hassona et al., who showed that pain prompts 73.5% of dental consultations [14]. Additionally, the most frequent indication for extraction was caries affecting either the adjacent second molar or the IMTM itself (20%), followed by recurrent pericoronitis in 17.5% of patients. This aligns with Adeyemo et al., who identified caries‐related pain as the main indication for third molar extraction (45.1%), followed by pericoronitis (22.4%) [15]. Partially erupted third molars caused caries in adjacent teeth (18.7%), in themselves (4.2%), or both (13.4%) [16]. Pentapati et al. reported caries on the distal surface of the mandibular second molar in 39.1% of erupted and 38.6% of IMTM, indicating that the risk persists even after eruption [17]. Other studies also identified recurrent pericoronitis as the leading extraction indication in 46.7% [13] and 44.6% of cases [18].

According to the American Association of Oral and Maxillofacial Surgeons, if there is insufficient anatomical space to accommodate normal eruption, removal of such teeth at an early age is valid and is a sound treatment rationale based on medical necessity [19]. Dentists recommend the removal of third molars for reasons not related to symptoms or pathology [10]. However, the National Institute for Clinical Excellence of England introduced guidelines relating to third molar surgery, advising against the prophylactic removal of third molars and listing specific indications for surgery [20]. Surgical removal of third molars in older patients carries a higher risk of postoperative complications, such as pain and delayed healing [21]. Therefore, PE is often recommended in younger patients, when healing is optimal and complication rates are lower [22]. The PE of lower third molars was performed in 26.7% of patients in this study, which might be performed less frequently than necessary, particularly in the possibility of developing unrecoverable caries in either the lower third molar itself or the adjacent tooth. Extracting the carious lower third molar could make the restoration of the affected lower second molar more feasible, which supports the PE of IMTM, particularly when considering that advancing age may significantly complicate the surgery. However, in several clinical situations, the indication for extraction of IMTM remains borderline and requires thorough patient consultation regarding the potential surgical risks and the implications of nonremoval. This study identifies a significant association between increasing age and higher DMFI scores (*p* = 0.002), which appears to influence the decision toward PE (*p* = 0.03). The incidence of caries in the lower third molar and the adjacent second molar was also significantly associated with age (*p* = 0.037). These findings suggest that age is a contributing factor to third molar pathology and may justify the preference among faculty members for recommending PE in a substantial proportion of cases. In this study, 26.7% patients were asymptomatic and presented for routine checkup, while 12.5% sought care due to esthetic concerns. This contrasts with the findings of Anyanechi et al., who reported that 84.1% of those who underwent third molar extraction were asymptomatic [23].

PE was justified by a good, thorough analysis of each case, particularly in asymptomatic patients or those with mild symptoms and poor eruption potential. About 56.3% of all PEs had IMTM near the second molars, posing a high risk of caries or root resorption, and 21.9% experienced difficulty with brushing, for which PE was recommended. These patients were considered at risk for developing caries or periodontal disease. Additionally, among the 13 patients with TMJ pain, five had nonerupting IMTM. Extraction was advised for these cases following consultation.

Age is a crucial factor in determining the difficulty scores for IMTM extraction, and it can complicate the surgery. Older adults frequently exhibit increased bone density, necessitating an increase in the amount of bone removal. The younger patients tend to experience less swelling and a quicker recovery due to the more favorable condition of their bone and root structures [24]. This study demonstrated a significant positive association between age and dental caries that supports the rationale for recommending PE of IMTM at earlier ages. This approach is especially justified when age and molar angulation indicate that eruption is unlikely to occur.

The risk of nerve injury is anticipated notably in patients aged 24–30, as impaction depth tends to increase with age, supporting the recommendation for removal at a younger age. In cases where the roots are near the nerve, retaining the tooth or opting for alternative procedures, such as coronectomy, may be considered [25]. This study showed a highly significant association between tooth angulation and the extraction decision (*p* < 0.001). Distoangular impactions were almost exclusively indicated for extraction due to recurrent pericoronitis and poor probability for eruption, whereas mesioangular and horizontal impactions were more frequently managed by extraction, including PE. Thus, 58% of mesioangular teeth were indicated for extraction, and 39% underwent PE. Among the affected teeth with caries in this study, the majority (41.7%) were mesioangular. Caries commonly develop on the occlusal or mesioproximal surfaces of partially or fully erupted lower third molars due to their inaccessible position, which hinders proper cleaning. Angulated impaction leads to food trapping and the slow development of distal cervical caries on the second molar. Patients often seek extraction at an older age when the caries are nonrestorable. Due to limited access and the risk of periapical or fascial space infections, extraction is recommended [26]. In the literature, a mesioangular that partially erupted mandibular third molar is associated with more caries in the adjacent tooth. PE of vertically and mesioangularly located third molars at an eruption level of Position A is recommended [27]. The position of the third molar may promote food impaction and create a favorable environment for caries evolution [26]. Tai et al. reported dental lesions in 24.63% and periodontal lesions in 35.3% of adjacent mandibular second molars. They also found that periodontal lesions were most common with mesioangular (48.81%), horizontal (17.31%), inverted (5.19%), and distoangular (10%) third molar impactions. Distal periodontal lesions on second molars were highest with mesioangular impactions (45.74%), distoangular (23.33%), horizontal (16.95%), and inverted (2.91%) [28].

Risk factors for developing systemic symptoms include mesioangular impaction, Class II ramus or Class B occlusal position, and smoking, which also increase the systemic symptoms [29]. In this study, 17.5% of patients showed signs of pericoronitis related to IMTM. Of these, 81% were scheduled for extraction due to the low likelihood of eruption, mainly based on age and molar angulation. Notably, 82.4% of these molars were mesioangular or distoangular, indicating bony impaction or contact with the adjacent second molar. The remaining four patients had vertically impacted molars; extraction was deferred due to young age and potential for eruption. These patients underwent operculectomy and were advised on proper oral hygiene.

Young individuals with good periodontal health clinically demonstrate periodontal pocketing, attachment loss, inflammatory, and pathogenic markers distal to second molars in the presence of third molars [30]. Periodontal lesions around IMTM were reported in 37.81% of patients, with 35.30% showing lesions on the distal aspect of adjacent second molars. The incidence of lesions varied by impaction angulation, with 48.81% in mesioangular impactions and 17.31% in horizontal impactions. For adjacent molars, distal periodontal lesions were observed in 45.74% of mesioangular, 16.95% of horizontal, and 23.33% of distoangular impactions [28] and 52% associated with deep periodontal pockets [31]. Wang et al. reported that 14.81% of second molars exhibited distal periodontal pathology leading to mobility and root resorption, while the frequency increases with age [32]. Mesioangular impactions showed a strong positive correlation between patient age and the severity of distal periodontal lesions in adjacent molars. Early prophylactic removal of mesioangular third molars and close monitoring of horizontally or invertedly impacted molars are recommended [28]. Additionally, extraction of vertically angulated soft tissue IMTM should be prophylactically considered [32]. In this study, periodontal lesions were observed in four patients; two had unerupted third molars with deferred extraction in hopes of eruption, while the other two underwent extraction due to unfavorable eruption prospects; both had horizontally impacted molars and were > 30 years.

Reports justify the prophylactic removal of asymptomatic, disease‐free IMTM, as it is thought to influence orthodontic treatment in various ways [33]. In this study, we recommended extraction in 11.7% of cases, half of which involved tooth buds, while extraction was deferred in 4% of patients. Anterior dental crowding is a common reason for orthodontic treatment [34]. Some studies suggest that third molars may contribute to mandibular crowding; the link remains unproven [34]. Husain and Rengalakshmi found higher Little′s index scores in patients with third molars compared to those without [35]. On the contrary, another study concludes that mandibular late incisor crowding is not significantly influenced by the presence of mandibular third molars or their development dynamics [36]. IMTM should be planned for early prophylactic removal in preparation for sagittal split osteotomies at least 6–12 months before the surgery [37]. Orthodontists advocate early removal to maintain a proper occlusion following orthodontic therapy, preventing late mandibular incisor relapse and irregularities. In contrast, some found no apparent connection and no evidence to support the preventative removal for reasons of occlusal stability [38]. Removal of the third molar before the development of pathology should be considered in patients with insufficient physiological space for eruption [2].

Surgical extraction of IMTM is challenging, which may contribute to the limited supporting evidence despite known complications. In this study, 24.2% of patients referred for extraction had no clear current or future indication, and the procedure was deferred. Many presented with symptoms unrelated to the impacted tooth. Analysis of deferred cases showed that the rate of extraction in 24% was declined after counseling about nerve injury risks, with patients opting against coronectomy. In 65.5%, extraction was postponed due to a good chance of natural eruption, following a wait‐and‐see approach, while 10.3% had fully impacted, but asymptomatic, so immediate removal was not deemed necessary. In several clinical situations, the indication for extraction of IMTM remains borderline and requires thorough patient consultation regarding the potential surgical risks and the implications of nonremoval. Performing extraction without a clear scientific or clinical justification may expose patients to unnecessary risks while also imposing avoidable demands on healthcare resources [39].

Patients with TMJ disorder are often misdiagnosed and unnecessarily advised to undergo IMTM extraction. In this study, 14.2% were initially suspected of having third molar‐related pain but were later diagnosed with TMJ disorder. Among them, extraction was deferred in 70.6%; however, 29.4% were consulted and underwent PE due to poor eruption potential or anticipated orthodontic needs, despite presenting with TMJ symptoms. The final diagnosis of patients presenting with TMD was established by an oral and maxillofacial specialist based on the TMD/DC criteria, including myofascial pain, clicking, and arthrogenic pain. The condition and its implications were thoroughly explained to the patients, who were subsequently referred to the Department of Medicine for management. This outcome aligns with Mackie et al., who found that 11.1% of patients referred for IMTM extraction were diagnosed with TMD, making surgery unnecessary [40]. Similarly, DeAngelis et al. reported that 13.3% of cases had pain from the TMJ and associated muscles [41]. Persistent lower facial pain is sometimes attributed to an IMTM discovered incidentally on routine radiographs. While this may prompt strong recommendations for extraction, it is essential to inform patients that removing the tooth may not resolve the pain. Also, the anatomical proximity of third molars to the TMJ can complicate the diagnosis and potentially lead to confusion with TMD pain [41].

The limitations of this study include a small sample size; however, the asymptomatic subgroup was likely sufficient for detecting moderate to large associations, and the absence of statistical significance in subgroup analyses should not be interpreted as evidence of the absence of association. Also, a single‐center design involving a regional population from Sulaymaniyah may limit the statistical power and generalizability of the findings and may require confirmation in larger multicenter studies. In addition, the endpoint of this study was recording the specialist′s final decision regarding extraction after consultation with the patient only. Finally, the study population consists of referred patients, which may result in a higher prevalence of pathology compared to the general population. Consequently, future studies with larger sample sizes, multicenter designs, and longer follow‐up periods evaluating postoperative outcomes are recommended to confirm these findings.

## 5. Conclusions

Based on the decision of a multidisciplinary group of faculty members, most of the referred patients had a clear indication for the extraction of the IMTM. The most frequent indication for extraction was caries involving either the adjacent second molar or the IMTM, followed by recurrent pericoronitis. Extraction of IMTM is particularly justified when factors, such as age and tooth angulation, suggest little or no potential for eruption. Management should prioritize patient quality of life, with decisions individualized according to the patient′s current and future health, social and financial circumstances, and risk tolerance. Prophylactic removal of asymptomatic IMTM may also be justified, as the incidence of caries increases with age. Many young individuals, despite being asymptomatic, seek dental evaluation due to increased health awareness. Nevertheless, in many cases, the indication for extraction remains borderline and requires careful consultation regarding surgical risks and the potential consequences of nonremoval.

## Author Contributions

S.N.A.: methodology, data collection, data analysis, study registration, and writing of the original manuscript; N.A.M.A.: conceptualization, supervision, methodology, and editing of the original manuscript.

## Funding

No funding was received for this manuscript.

## Disclosure

Both authors agreed to submit the manuscript to this journal.

## Ethics Statement

The study protocol was reviewed and approved by the Ethical Committee of the College of Dentistry, University of Sulaimani, Sulaymaniyah, Iraq (No. 206/23 dated November 23, 2023). The study was conducted in accordance with the Declaration of Helsinki. Patients′ written informed consent was obtained prior to the research, and they were free to withdraw at any time.

## Conflicts of Interest

The authors declare no conflicts of interest.

## Data Availability

The data that support the findings of this study are available on request from the corresponding author. The data are not publicly available due to privacy or ethical restrictions.
